# Resolving a contradiction: full gene deletion of *kqt-3* by CRISPR does not lead to pharyngeal pumping defects

**DOI:** 10.17912/micropub.biology.000137

**Published:** 2019-07-25

**Authors:** Rosemary Bauer, Benjamin Nebenfuehr, Andy Golden

**Affiliations:** 1 Laboratory of Biochemistry and Genetics, National Institute of Diabetes and Digestive and Kidney Diseases, NIH, Bethesda, MD 20892; 2 Current address: Department of Molecular, Cellular, and Developmental Biology, University of Colorado Boulder, Boulder, CO 80303

**Figure 1:**
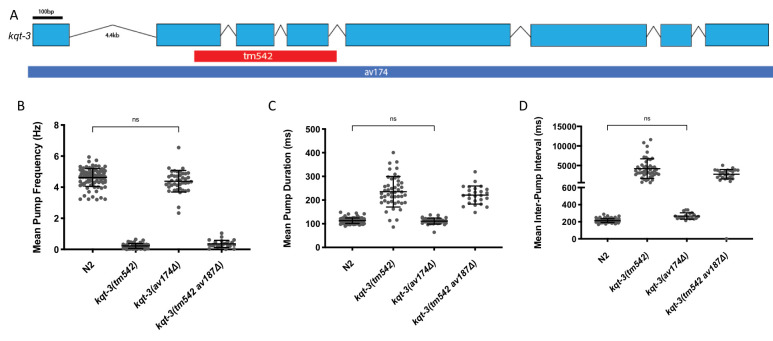
(A) *kqt-3* is 8869bp long and encodes KQT-3, a voltage-gated potassium channel. The *tm542* deletion removes 1001bp beginning in exon 2 and ending within intron 4. The *av174* deletion removes 9004bp beginning 69bp upstream of the ATG and ending 66bp after the stop (*av174Δ*). (B-D) The full length deletion of *kqt-3* does not mimic the smaller deletion allele, suggesting defects in (B) pump frequency, (C) duration, and (D) inter-pump interval are not due to *kqt-3* mutations. *kqt-3(tm542)* mutants differ greatly from WT in all three measures (one way ANOVA, Tukey’s multiple comparisons test, p<0.001), but *kqt-3(av174Δ)* mutants do not (one way ANOVA, Tukey’s multiple comparisons test, ns). Creation of the full-length deletion in the *kqt-3(tm542)* background, notated *kqt-3(tm542av187Δ)*, does not rescue the pumping defects observed in the *tm542* mutant. Introns not to scale. Bars represent mean ± 1 SD. Average n=53.

## Description

KQT-3 is the *C. elegans* ortholog of human KCNQ1, a voltage-gated potassium channel that is implicated in multiple types of Long Q-T cardiac arrhythmias including Jervell and Lange-Nielsen syndrome and Romano-Ward syndrome. In order to assess the possibility of modeling these syndromes in *C. elegans*, we obtained the *tm542* deletion allele, which had previously been associated with changes to the defecation cycle (Nehrke et al., 2008, Kwan et al. 2008). Any visible, easily-scored phenotype would be an indication that we could move forward with creating patient-specific missense alleles by CRISPR.

The *tm542* deletion, a 1001bp deletion generated by chemical mutagenesis that disrupts exons 2-4, appeared to cause a dramatic reduction in pharyngeal pump frequency (Fig 1B). This was an indication that mutations to *kqt-3* might be a reasonable way to model Long Q-T arrhythmias in worms. When three patient missense mutations and two smaller deletions yielded only very subtle changes to pharyngeal pumping, we became increasingly suspicious of the *tm542* deletion strain. This prompted the creation of a full-length, 9004bp deletion allele by CRISPR that starts 69bp upstream of the start codon, ends 66bp after the stop codon, and inserts stop codons in every frame. Surprisingly, complete deletion of this 621 amino acid protein led to virtually no change in pharyngeal pumping (Fig 1B-D).

In order to confirm that the pharyngeal pumping phenotype in the *kqt-3(tm542)* strain was due to extraneous mutations and not a dominant negative effect, we created the full-length deletion of *kqt-3* by CRISPR in the *tm542* strain. This *kqt-3(tm542av187Δ)* deletion strain phenocopied the original *tm542* deletion (Fig 1B-D), indicating that other mutations besides the deletion in *kqt-3* are responsible for the lack of pharyngeal pumping in the *tm542* strain.

This study should serve as a cautionary tale for any lab studying a deletion allele that was generated by chemical mutagenesis; the strain likely contains numerous other mutations. We now routinely generate a full gene deletion by CRISPR as part of any genetic study.

## Reagents

Strains

FX542 *kqt-3(tm542)* II AG451 *kqt-3(av174∆)* II AG479 *kqt-3(tm542av187∆)* II

Genotyping

Oligos used for genotyping *tm542*: expect sizes 1342bp WT and 340bp *tm542*. F: 5’-ttccggtaagaaacttctgg R: 5’-cccaaaactccaccaattagc

Oligos used for genotyping *av174∆* and *av187∆*: one internal pair and one external pair; expect sizes 716bp WT from internal reaction and 486bp full gene deletion from external reaction.

F internal: 5’-cgccacaaatttttgtgtcc R internal: 5’-ggtcagaaatgttgcggaat F external: 5’-gctcattctctctcacaccac R external: 5’-cccaaatcatgttgacagcgag

Electropharyngeograms

15 gravid hermaphrodites were placed on OP50-seeded MYOB plates for 6 hours to establish a synchronized population. After 3 days the population was washed off with M9, washed 3x, then incubated in 10mM 5-HT for at least 20 minutes. Pharyngeal pumping was then recorded using the NemaMetrix ScreenChip system (Eugene, OR). All strains were recorded on at least two separate days. Outliers were identified by ROUT with Q=0.5%. Strains were compared by one-way ANOVA with Tukey’s multiple comparison test.

CRISPR/Cas9

The *av174* and *av187* deletions were created using clone-free CRISPR-mediated gene editing (Paix et al. 2015) with *dpy-10* co-CRISPR (Arribere et al. 2014). The deletion was generated using crRNAs at both the 5’ and 3’ ends of *kqt-3* (5’: 5’-actagtagtaatatattaaa; 3’: 5’-gaccacgacgcatcaacagc) and a single-stranded repair template containing an insertion of stop codons in every frame (TAGATAAATGA) and 35bp of homology on both sides. This mix was injected into N2 to obtain *kqt-3(av174∆)* and into *kqt-3(tm542)* to obtain *kqt-3(tm542av187∆)*.
